# Arbuscular mycorrhizal colonization does not improve root hydraulic supply in tomato and pea

**DOI:** 10.1093/plphys/kiaf669

**Published:** 2025-12-23

**Authors:** Jiacan Sun, Timothy J Brodribb, Eloise Foo, Ibrahim Bourbia

**Affiliations:** Discipline of Biological Sciences, School of Natural Sciences, University of Tasmania, Private Bag 55, Hobart, Tasmania 7001, Australia; Discipline of Biological Sciences, School of Natural Sciences, University of Tasmania, Private Bag 55, Hobart, Tasmania 7001, Australia; Discipline of Biological Sciences, School of Natural Sciences, University of Tasmania, Private Bag 55, Hobart, Tasmania 7001, Australia; Discipline of Biological Sciences, School of Natural Sciences, University of Tasmania, Private Bag 55, Hobart, Tasmania 7001, Australia

## Abstract

Arbuscular mycorrhizal (AM) fungi are known to enhance plant drought tolerance, but the physiological mechanism behind this benefit remains unclear. One explanation is that AM colonization improves root hydraulic conductance (*K*_r_), thereby facilitating more efficient water uptake under soil drying, though this mechanism remains highly debated. Here, we measured *K*_r_ in tomato (*Solanum lycopersi*cum L.) and pea (*Pisum sativum* L.) with and without AM using a noninvasive rehydration technique under soil drying, and this was complemented with the evaporative flux method under hydrated conditions. AM colonization was manipulated either through soil sterilization or by using nonmycorrhizal mutants, ensuring precise control of AM status. In both species, AM colonization had no positive impact on *K*_r_ under both well-hydrated and drought conditions. The finding suggests that the improved drought performance often observed in AM-colonized plants is not due to enhanced root water transport capacity. Instead, AM-induced benefits under drought may be mediated by other physiological adjustments.

## Teaser

Arbuscular mycorrhizal symbiosis had no effect on root hydraulic conductance in 2 major crops, suggesting it does not improve drought tolerance by increasing water uptake.

## Introduction

Roots play a major role in supplying the shoot canopy with water, maintaining productivity and survival. Root water uptake capacity has been reported to be highly sensitive to the initial stages of soil drying, reducing plant water uptake and gas exchange (Rodriguez-Dominguez and Brodribb 2020; Bourbia et al. 2021). Root hydraulic conductance (*K*_r_) reflects the efficiency of plant roots to conduct water from the root–soil interface to the root collar (Steudle 2000). The dynamics of *K*_r_ during soil drying have been shown to be influenced by several factors such as root structure, root length, and aquaporin activity (Vadez 2014; Jeanguenin et al. 2016). However, the role of rhizosphere microorganisms on *K*_r_ dynamics under progressive soil drying is still poorly understood.

Rhizosphere microorganisms play an important role in plant growth, health, and ecosystem sustainability. This includes beneficial microbes that promote plant nutrient uptake, as well as detrimental microbes that challenge plant health (Mendes et al. 2013; Omotayo and Babalola 2021). Arbuscular mycorrhizal fungi (AMF) belong to the phylum Glomeromycota and form symbioses with 71% of all land plants (Brundrett and Tedersoo 2018). Arbuscular mycorrhizal (AM) symbioses promote plant nutrient uptake (Wahab et al. 2023), plant growth, and, in many cases, drought tolerance (Yan et al. 2022; Obase et al. 2023). While the role of AM associations in the uptake of nutrients is well understood, the ability of AM-colonized plants to enhance water uptake under soil drying conditions is still a matter of debate (reviewed by Abdalla et al. 2023).

In addition to root hydraulics, plants employ a range of adaptive strategies to resist drought stress. These include hormonal regulation, such as abscisic acid (ABA)-mediated stomatal closure and the modulation of other hormone pathways; osmotic adjustment through the accumulation of osmolytes like proline, sugars; and morphological modifications such as increased root length (Fang and Xiong 2015; Lim et al. 2015; Kaur et al. 2016; Ruiz-Lozano et al. 2016; Naikwade 2023). These mechanisms help to preserve cellular hydration and maintain productivity under water stress. AM symbiosis has been proposed to influence some of these processes, for example, by modulating ABA signaling or improving soluble sugar contents that support osmotic adjustment, thereby contributing to plant drought tolerance through multiple mechanisms (Xu et al. 2018; Yang et al. 2025).

Plants with AM symbiosis have been reported to exhibit higher photosynthesis, stomatal conductance (*g*_sc_), and transpiration compared to corresponding nonmycorrhizal plants under both well hydrated and drought conditions (Kuyper et al. 2021). For example, a meta-analysis conducted by Augé et al. (2016), integrating findings from 1,019 studies, reported that AM-colonized plants exhibited a 49% increase in carbon exchange rate, along with a 28% and 26% rise in *g*_sc_ and transpiration, respectively, compared to non-AM plants. Similarly, a study conducted by Kakouridis et al. (2022) showed that oats with AMF transpired twice as much as those without, indicating that AMF may extend the roots access to water. The higher *g*_sc_ of AM plants during soil drying suggested that AMF may access water unavailable to non-AM plants, potentially by the hyphal networks taking up water (Kakouridis et al. 2022). While studies have highlighted the positive effects of AMF on aboveground physiological function, less attention has been paid to their influence on the function of root hydraulics under drought conditions.

One possible explanation for the enhanced drought tolerance of AM-colonized plants is an increase in *K*_r_. Ideally, this hypothesis should be tested using a direct, noninvasive method to measure *K*_r_ continuously from well-hydrated to drought conditions. While previous studies have examined the impact of AM colonization on root hydraulics, their results have varied with some reporting increased *K*_r_, others showing no effects on *K*_r_ (Allen 1982; Levy et al. 1983; Graham and Syvertsen 1984; Gonzalez-Dugo 2010; Abdalla and Ahmed 2021; Pauwels et al. 2023). The discrepancy remains elusive and may stem from differences in species-specific behaviors or experimental design and measurement approaches used to examine *K*_r_. For example, in many cases *K*_r_ is measured indirectly using the evaporative flux method, which assumes steady-state plant water potential and transpiration (Gonzalez-Dugo 2010; Abdalla and Ahmed 2021). The steady-state assumption may not hold during progressive soil drying, as plant and soil water potentials as well as transpiration continuously decline throughout the day (Bourbia et al. 2022; Bourbia et al. 2025). Other techniques involve invasive approaches, such as the root pressure probe and the pressure chamber (Knipfer and Steudle 2008 ), provide valuable insights but involve steps that may alter root functions or introduce errors due to root damage and do not reflect natural conditions (Li and Liu 2010). Furthermore, these studies focused on well-hydrated conditions or compared only extreme wet and dry states (reviewed by Kakouridis et al. 2022), without examining a progressive decline in soil moisture where plants actively regulate water use and carbon gain.

To address these limitations and provide robust evidence for the influence of AM symbiosis on root water transport under realistic drought scenarios, we use a noninvasive rehydration method for directly measuring *K*_r_ based on the relaxation kinetics of stem water potential during hydration of plants from different levels of soil water stress (Rodriguez-Dominguez and Brodribb 2020; Bourbia et al. 2021). By applying this noninvasive rehydration method, *K*_r_ dynamics in AM and non-AM plants were compared. To clarify the effects of AM, we combined 2 complementary strategies to control AMF: inoculation with active or sterilized AMF inoculum, and the use of nonmycorrhizal mutants. The study was conducted with 2 herbaceous species, *Solanum lycopersicum* L. (tomato) and *Pisum sativum* L. (pea), under a large range of soil water stress, from well-hydrated conditions to moderate stress that was sufficient to cause a substantial decline in *K*_r_. This combination of a noninvasive rehydration method, 2 AMF control strategies and multiple cultivars of tomato and pea provides an ideal framework for understanding how AM symbiosis influences root hydraulics during dehydration and recovery.

## Results

### AM colonization has no impact on *K*_r_ in wild-type tomato under hydrated or drought conditions

**Figure 1.**
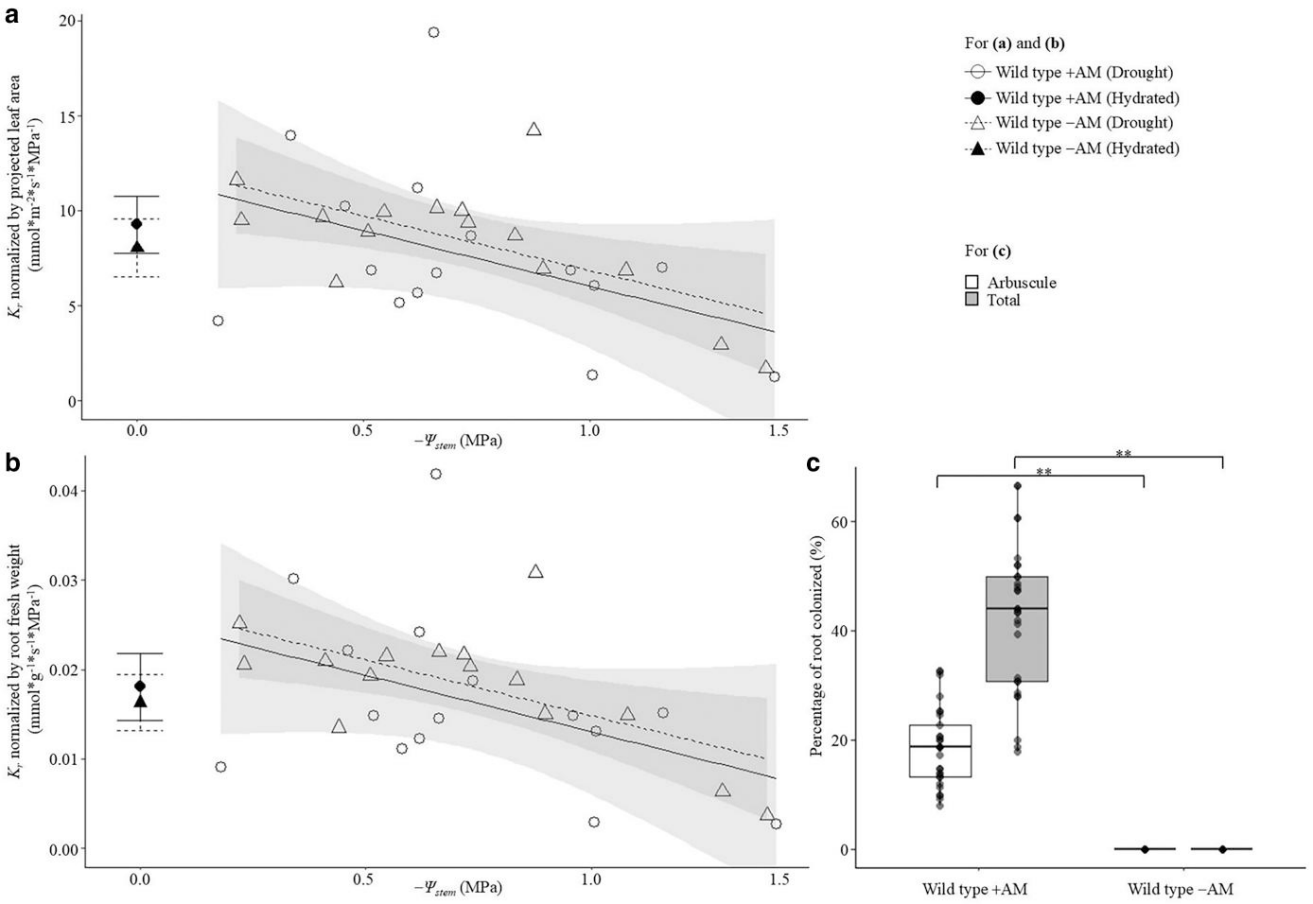
Root hydraulic conductance (*K*_r_) in *Solanum lycopersicum* L. wild type (M82) with arbuscular mycorrhiza (+AM) and without arbuscular mycorrhiza (−AM). (a and b) Scatter plot showing *K*_r_ normalized by (a) projected leaf area and (b) normalized by root fresh weight under hydrated and drought conditions (−Ψ_stem_ = 0–1.5 MPa). The shaded regions represent confidence intervals (95%) for each treatment group, with the fitted lines indicating the trend of *K*_r_ under drought conditions in relation to −Ψ_stem_. Large error bars at *−*Ψ_stem_ = 0 indicate mean ± standard error under hydrated conditions; solid and dashed lines represent wild-type +AM and wild-type −AM treatments, respectively; (c) percentage of root colonized by AM. The center line represents the median; box limits indicate the upper and lower quartiles; whiskers extend to 1.5× the interquartile range; points represent individual biological replicates, with points beyond the whiskers considered outliers. For (a–c), data points represent individual replicate samples. Asterisks indicate significant differences between treatments (*t*-test, **P < 0.01). The sample size of M82 ± AM under drought or hydrated conditions is *n* = 14 to 15.

The relationship between *K*_r_ normalized by projected leaf area (Fig. 1a) or root fresh weight (Fig. 1b) and Ψ_stem_ under different water stress conditions was observed in wild-type tomato (cultivar M82) colonized with arbuscular mycorrhiza (+AM) and without arbuscular mycorrhiza (−AM). Successful AM colonization occurred in +AM inoculated tomato plants and was zero in −AM plants (Fig. 1c). There was no significant difference between wild-type tomato plants +AM and −AM for leaf area, shoot and root weight, or the ratio of shoot-to-root (Supplementary Figure S1).

*K*_r_ decreased linearly in tomato with declining Ψ_stem_ in all treatments, whether *K*_r_ was normalized by projected leaf area or root fresh weight (Fig. 1a, b). Under hydrated conditions (Ψ_stem_ = 0 MPa), there was no significant difference in *K*_r_ in wild-type tomato plants colonized with AM fungi or not colonized with AM fungi (*t*-test: *P*-value = 0.571/0.839 for *K*_r_ under hydrated conditions normalized by projected leaf area or root fresh weight; Fig. 1a, b). Similarly, across a wide range of Ψ_stem_, the presence or absence of AM colonization had no significant impact on *K*_r_ dynamics during soil drying (Fig. 1a, b; statistical analysis using the MuMIn package in R, with model selection result from Equation (3): ΔAICc = 0.00/0.00 in Model 1 and 3, respectively, Supplementary Table S1).

### *K*_r_ is similar in AM-colonized wild-type tomato and nonmycorrhizal mutant *rmc* under hydrated and drought conditions

**Figure 2.**
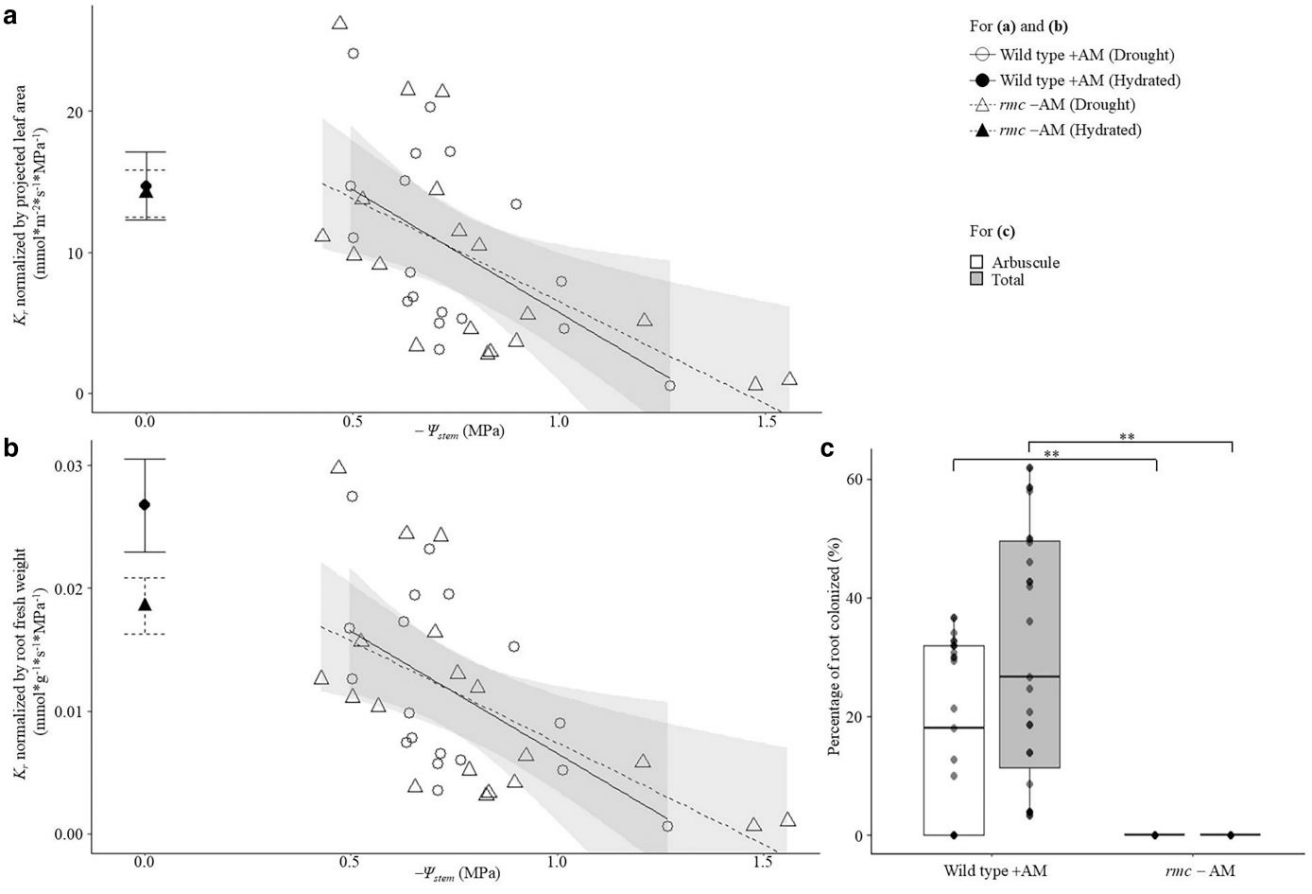
Root hydraulic conductance (*K*_r_) in *Solanum lycopersicum* L. wild type (76R) colonized by mycorrhizal fungi (+AM) and respective nonmycorrhizal mutant *rmc* (*−*AM). (a and b) Scatter plot showing *K*_r_ normalized by (a) projected leaf area and (b) normalized by root fresh weight under hydrated and drought conditions (*−*Ψ_stem_ = 0–1.5 MPa). The shaded regions represent the confidence intervals (95%) for each treatment group, with the fitted lines indicating the trend of *K*_r_ under drought conditions in relation to *−*Ψ_stem_. Large error bars at *−*Ψ_stem_ = 0 indicate mean ± standard error under hydrated conditions; solid and dashed lines represent wild-type +AM and mutant *rmc* −AM, respectively; (c) percentage of root colonized by AM. The center line represents the median; box limits indicate the upper and lower quartiles; whiskers extend to 1.5× the interquartile range; points represent individual biological replicates, with points beyond the whiskers considered outliers. For (a–c), data points represent individual replicate samples. Asterisks indicate significant differences between treatments (*t*-test, **P < 0.01). The sample size of 76R and *rmc* under drought or hydrated conditions is *n* = 9–19.

To validate the findings from the experiment comparing +AM and −AM in wild-type tomato cultivar M82 using the sterilization method (Fig. 1), the wild-type tomato cultivar 76R and its nonmycorrhizal mutant *rmc* were tested. As expected, *rmc* could not establish AM symbiosis and displayed no AM colonization, while wild-type 76R was well colonized (Fig. 2c). There was no significant difference between wild-type 76R and *rmc* tomato plants in leaf area, shoot and root weight, or the ratio of shoot-to-root (Supplementary Figure S1). The *rmc* mutant plants exhibited similar *K*_r_ dynamics in response the decline in Ψ_stem_ from −0 to −1.5 MPa as observed in wild-type plants (Fig. 2a, b; ΔAICc = 0.00/0.00 in Model 5 and 7, respectively, Supplementary Table S1). Under well-watered conditions (Ψ_stem_ = 0 MPa), no significant differences in *K*_r_ were observed between the 2 genotypes (*t*-test: *P*-value = 0.862/0.111 for *K*_r_ under hydrated conditions normalized by projected leaf area or root fresh weight; Fig. 2a, b).

### *K*_r_ is similar in AM-colonized wild-type pea and nonmycorrhizal mutant *sym19* under hydrated and drought conditions

**Figure 3.**
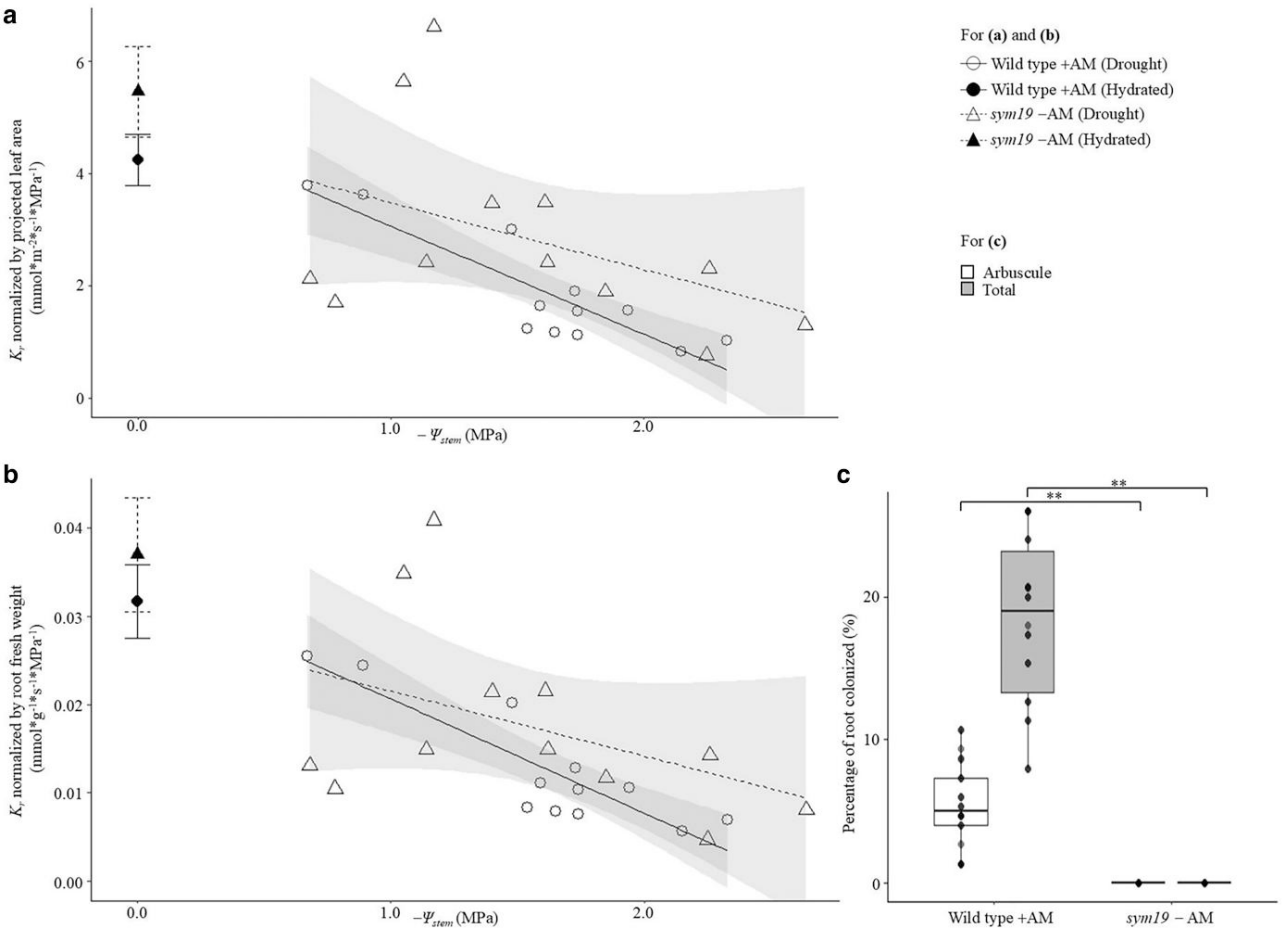
Root hydraulic conductance (*K*_r_) in *Pisum sativum* L. wild-type (Frisson) and respective nonmycorrhizal mutant *sym19* (*−*AM). (a and b) Scatter plot showing *K*_r_ normalized by (a) projected leaf area and (b) normalized by root fresh weight under hydrated and drought conditions (*−*Ψ_stem_ = 0–2.5 MPa). The shaded regions represent the confidence intervals (95%) for each treatment group, with the fitted lines indicating the trend of *K*_r_ under drought conditions in relation to *−*Ψ_stem_. Large error bars at *−*Ψ_stem_ = 0 indicate mean ± standard error under hydrated conditions; solid and dashed lines represent wild-type +AM and mutant *sym19* −AM, respectively; (c) percentage of root colonized by AM. The center line represents the median; box limits indicate the upper and lower quartiles; whiskers extend to 1.5× the interquartile range; points represent individual biological replicates, with points beyond the whiskers considered outliers. For (a–c), data points represent individual replicate samples. Asterisks indicate significant differences between treatments (*t*-test, **P < 0.01). The sample size of Frisson and *sym19* under drought or hydrated conditions is *n* = 11–13.

To investigate the impact of AM symbiosis on *K*_r_ in pea, wild-type cultivar Frisson was compared with its nonmycorrhizal mutant *sym19* under both hydrated and drought conditions. Due to the mutation, *sym19* is unable to establish AM symbiosis and displays zero AM colonization (Fig. 3c). In pea, AM symbiosis did not have a positive impact on *K*_r_. Under hydrated conditions, *K*_r_ was not significantly different between wild-type pea and nonmycorrhizal *sym19* mutant plants (*t*-test: *P*-value = 0.206/0.494 for *K*_r_ under hydrated conditions normalized by projected leaf area or root fresh weight; Fig. 3a, b). Interestingly, under drought conditions, a small but significant increase in *K*_r_ was observed in the nonmycorrhizal mutant *sym19* compared to the AM-colonized wild-type pea cultivar (Fig. 3a, b). This was the case if *K*_r_ was normalized by projected leaf area or root fresh weight (ΔAICc = 0.00/0.27/0.00/0.90 in Model 9, 10, 12, and 13, respectively, Supplementary Table S1). It is important to note that the shoot fresh weight of *sym19* was significantly lower than wild type (*P* < 0.01), despite similar root sizes (Supplementary Figure S1). Thus, it is possible that the small increase in *K*_r_ in the nonmycorrhizal mutant *sym19* might be caused by smaller shoot size compared to wild-type pea.

### The relationship between AM root colonization and *K*_r_

**Figure 4.**
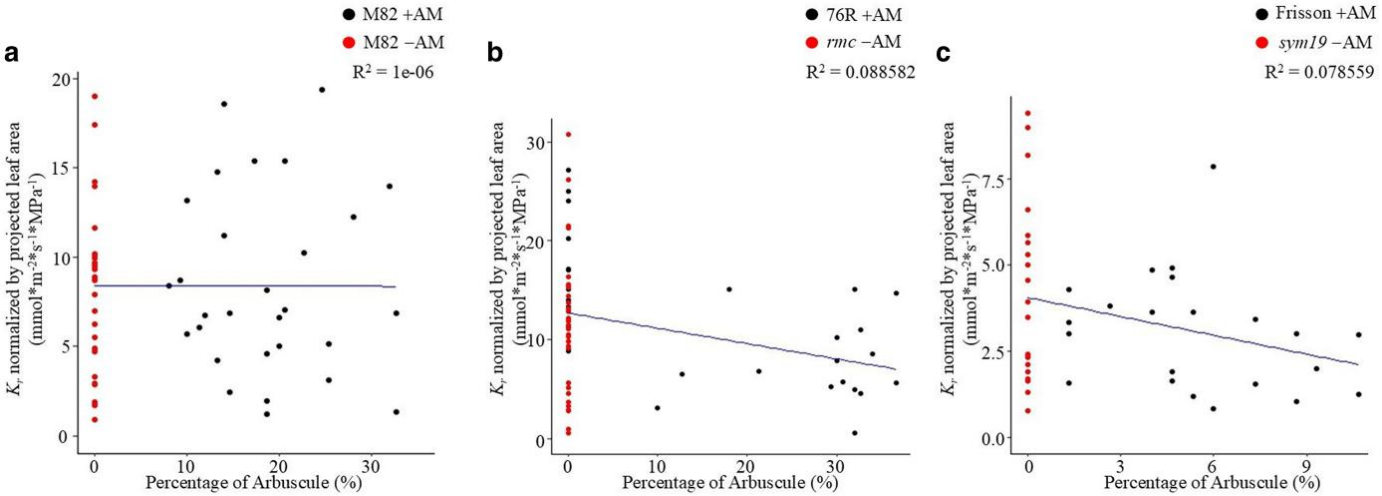
Relationship between the percentage of the root colonized by arbuscules and root hydraulic conductance (*K*_r_) normalized by projected leaf area across 3 genotypes in *Solanum lycopersicum* L. and *Pisum sativum* L. (a) Tomato M82 (wild type), with arbuscular mycorrhiza (+AM) and without arbuscular mycorrhiza (−AM). (b) Tomato 76R (wild type) and its nonmycorrhizal mutant, *rmc*. (c) Pea Frisson (wild type) and its nonmycorrhizal mutant, *sym19*. *R*² values are displayed in the top-right corner to indicate the goodness of fit for each regression model.

To determine if there was any relationship between the extent of the root colonized by AM fungi and *K*_r_, the percentage of root colonization by arbuscules was compared to *K*_r_ normalized by projected leaf area across different genotypes in tomato and pea (Fig. 4). No correlation between the percentage of the root colonized by arbuscules and *K*_r_ normalized by projected leaf area was found across both wild-type lines of tomato (M82 and 76R). In the tomato wild-type M82, no significant correlation was found between the percentage of arbuscule colonization and *K*_r_, as indicated by the *R*^2^ value close to zero (*R*^2^ = 1e−06; Fig. 4a). Both M82 plants colonized by AM (+AM) and not colonized due to soil sterilization (−AM) displayed a wide distribution of *K*_r_, with no obvious trend that could be explained by the level of arbuscule colonization. In the wild-type tomato 76R and respective nonmycorrhizal mutant *rmc*, no *rmc* plants were colonized and at least some individual wild-type 76R plants displayed low levels of colonization, but there was no evidence of a link between the percentage of the root colonized by arbuscules and normalized *K*_r_, with a low *R*^2^ value of 0.088582 (Fig. 4b). Similarly, no correlation between arbuscule colonization and normalized *K*_r_ was found in the wild-type pea Frisson and respective nonmycorrhizal mutant *sym19*, with an *R*^2^ value of 0.078559 (Fig. 4c).

There was also no relationship found between *K*_r_ normalized by projected leaf area and total root colonization (the percentage of root colonized by any fungal structure; arbuscules, hyphae, and vesicles) across tomato or pea (Supplementary Figure S2). Similarly, no relationship was found between *K*_r_ normalized by root fresh weight and the percentage of root colonization by arbuscules (Supplementary Figure S3) or by total colonization (Supplementary Figure S4).

### AM symbiosis does not influence *C*_plant_ in pea or tomato

To investigate whether AM symbiosis influences whole-plant capacitance, *C*_plant_, we compared *C*_plant_ between plants with and without AM symbiosis across different species and genotypes. We hypothesized that AM symbiosis might enhance the plant's capability to hold water, causing an increase in *C*_plant_. However, no significant differences were observed in *C*_plant_ between +AM and −AM for any genotype (Supplementary Figure S5), suggesting that the presence of AM symbiosis did not substantially influence *C*_plant_ in pea or tomato. For wild-type tomato cultivar M82, there was no significant change in *C*_plant_ when the +AM and −AM conditions were compared (Supplementary Figure S5). For nonmycorrhizal mutants, *rmc* and *sym19*, which could not form AM symbiosis even in the presence of AMF, no differences in *C*_plant_ were found in these mutants and respective wild types, 76R and Frisson (Supplementary Figure S5). These consistent results across species suggested that AM symbiosis did not contribute to *C*_plant_, whether normalized by leaf area or root fresh weight, under the conditions tested.

## Discussion

AMF are widely recognized for their ability to enhance plant drought tolerance (Ruiz-Lozano et al. 1995; Boomsma and Vyn 2008; Das and Sarkar 2024). However, the direct evidence linking AM colonization to increased *K*_r_ remains ambiguous. In previous studies, AM effects on *K*_r_ were typically examined under either wet or severely dry conditions, with little focus on the intermediate range where plants actively regulate water uptake. In this study, we integrated genetic and sterilization approaches across multiple cultivars of tomato and pea, to assess the influence of AM on *K*_r_ using a noninvasive rehydration method across a wide range of soil moisture conditions. By using a highly controlled experimental design, our study provides consistent and definitive evidence that AM did not significantly affect *K*_r_ in either tomato or pea under either hydrated and drought conditions. Thus, the commonly observed drought tolerance in AM plants is likely due to indirect physiological changes facilitated by AM rather than increased root water uptake efficiency.

It is worth pointing out that *K*_r_ can be influenced by traits such as root mass, root surface area, and root-to-shoot ratio, which can pose a challenge when comparing AM and non-AM plants, as non-AM plants are frequently smaller due to limited nutrient uptake. However, in our tomato studies, no differences were observed in leaf area, root mass, or root-to-shoot ratio between AMF and non-AM plants, allowing for a direct comparison of *K*_r_ between the 2 groups. Interestingly, a higher *K*_r_ normalized by projected leaf area and root fresh weight was observed in nonmycorrhizal pea mutant *sym19* compared to wild-type pea cultivar Frisson under drought conditions (Fig. 3a, b, Supplementary Table S1: Model 9, 10, 12, and 13). This suggested AM symbiosis not only failed to enhance *K*_r_ under drought stress but also resulted in slightly lower *K*_r_ compared to the non-AM mutant *sym19*. The physiological data (Supplementary Figure S1a, b) showed significant reduction in projected leaf area and shoot fresh weight in *sym19* compared to wild-type Frisson, while the root fresh weight and ratio of shoot and root remained unchanged (Supplementary Figure S1c, d). Nonmycorrhizal mutant *sym19*, compared to the wild-type Frisson, failed to establish symbiosis with AMF (Albrecht et al. 1999), possibly leading to reduced nutrient uptake. The smaller size of aboveground tissue may have reduced the water demand in *sym19*, indirectly contributing to its higher *K*_r_. Indeed, other studies have found that AM colonization influences shoot biomass (Zhang et al. 2011; Qin et al. 2022). This suggests that *K*_r_ in pea was influenced by aboveground traits, rather than changes in the root system or AMF colonization. Importantly, AM symbiosis clearly did not improve *K*_r_ in pea.

AM colonization has been suggested to modify soil water retention properties and soil–root hydraulic interactions, potentially through altering the hydraulic conductance of the soil by binding the soil to the root surface, reducing the loss of hydraulic conductivity at the root–soil interface (Miller and Jastrow 2000; Bitterlich et al. 2018a). However, our results clearly demonstrate that any such influence does not extend significantly to internal root hydraulic conductance (the conductance from the root surface to the xylem). Instead, AM-mediated changes in soil properties may help maintain water flow from the bulk soil to the root surface, thereby sustaining some level of transpiration during drying, even when *K*_r_ is low in AM plants. This could explain the higher transpiration rates and drought tolerance often reported in AM plants under drying conditions (Bitterlich et al. 2018b).

The expression of aquaporins, which facilitate water movement across cell membranes and play a key role in regulating root water transport capacity, especially under water-limited conditions (Prado and Maurel 2013; Maurel et al. 2015), has become recognized as a major determinant of *K*_r_. If AMF colonization upregulates the activity of aquaporins, an increase in *K*_r_ would be expected in colonized plants. However, the fact that *K*_r_ was not increased by AM colonization in our experiments suggests that aquaporin-mediated water transport was not significantly upregulated in pea or tomato AM plants. In fact, under drought, AM plants may downregulate aquaporins to reduce water loss (Zou et al. 2019). In addition to aquaporins, we can rule out AM-induced changes in root anatomical traits such as suberization of the endodermis, xylem development, and radial resistance as they would also influence *K*_r_ (Steudle 2000). Increased suberization under drought can reduce apoplastic flow and thereby limit *K*_r_ (Enstone et al. 2002; Franke and Schreiber 2007; McLean et al. 2011), regardless of AM colonization.

Labeled water uptake has been used to understand the contribution of AM hyphae to root water supply. Kakouridis et al. (2022) used a 2-chamber system in oats to track water transport by fungal hyphae, using ¹⁸O-labeled water as well as a fluorescent dye. They found that fungal hyphae directly transported 34.6% of transpired water via the extracellular pathway. In contrast, Püschel et al. (2020) used the same methodological approach in *Medicago truncatula* and found that water transported by AMF hyphae was relatively small compared to the plant's overall water demands. Although these and other studies (eg Khalvati et al. 2005 ) have shown the potential for AMF hyphae to take up and transport water, the small diameter of the hyphae imposes biophysical limitations on volume flow, making their direct contribution to *K*_r_ likely negligible under well-hydrated conditions and during soil drying (Rengel and Djalovic 2021). Our results clearly indicate that any direct contribution of AMF hyphae to internal *K*_r_ is negligible.

Although our results indicate that AMF did not significantly alter *K*_r_ in either tomato or pea, other studies have reported contrasting outcomes linked to AMF genotype and stress severity (Chitarra et al. 2016; Ruiz-Lozano et al. 2016). Such variability makes cross-study comparisons difficult. In our study, however, the same AMF strains and plant varieties were used within each species, thereby minimizing variation due to plant and fungal genetic background. Regarding drought intensity, the stress treatments applied covered a range of water potentials, which allowed compare responses across moderate to severe stress levels.

Our study suggests the enhanced drought tolerance frequently reported in AM-colonized plants (Zhang et al. 2019; Huang et al. 2020; Yang et al. 2025) is not due to increased *K*_r_. Alternative explanations for AM-induced drought tolerance should be considered. Among the possible mechanisms, hormonal regulation and improved nutrient acquisition are likely key factors. AMF-induced modulation of hormones has been associated with improved performance of AM plants under drought compared to non-AM plants (reviewed by Cheng et al. 2021), such as strigolactones and ABA (Ruiz-Lozano et al. 2016). In addition, improved nutrient acquisition in AM plants, particularly phosphorus, could facilitate osmotic adjustment and metabolic balance during drought compared to non-AM plants (Püschel et al. 2021; Ullah et al. 2022). Supporting this view, some transcriptomic studies have shown that AM symbiosis modulates drought-responsive genes in host roots, including those involved in hormone signaling, osmotic adjustment, and secondary metabolite pathways (Balestrini et al. 2019; Xie et al. 2023, reviewed by Wang et al. 2023). This highlights the multiple mechanisms by which AM symbiosis can contribute to plant drought tolerance.

## Conclusion

Using a robust experimental design, our study shows that AMF does not enhance *K*_r_ under either hydrated or drought conditions in tomato and pea. This is significant because it challenges the assumption that AMF improves drought tolerance primarily through enhanced *K*_r_. Instead, our data indicate that in the distantly related species tomato and pea that AM did not influence *K*_r_ and it is likely that this is likely to also occur in other angiosperms. This suggests that the improved drought tolerance observed in AM-colonized plants is not driven by changes in *K*_r_. AMF-induced drought tolerance likely depends on a range of mechanisms, including hormonal regulation and nutrient uptake.

## Materials and methods

### Plant material and growth conditions

Tomato (*S. lycopersicum* L.) and pea (*P. sativum* L.) were used in the experiments. Two wild-type genotypes of tomato M82 and 76R cultivar were used, along with the corresponding reduced AM colonization mutant (*rmc*) on 76R background. M82 and 76R formed AM symbioses in the presence of AM fungi, while *rmc* is the nonmycorrhizal mutant that has a large deletion that contains the *CYCLOPS/IPD3* gene (Prihatna et al. 2018). The CYCLOPS/IPD3 protein plays a crucial role in the symbiotic signaling pathway, regulating the transcription of genes essential for establishing and functioning of AM symbioses (Yano et al. 2008). Tomato seeds were germinated in potting mix in punnet pots and transplanted after 3 wk in 0.5-liter (L) pots containing a substrate mixture of gravel, vermiculite, and live mycorrhizal inoculum; with a ratio of 2:2:1 or 2:2:2. The mycorrhizal inoculum used in this study came from the living corn (*Zea mays*) pot culture inoculated with the spores of *Rhizophagus irregularis* (INOQ Advantage, INOQ GMBH, Germany). Corn was planted in the substrate with 2-L pots (gravel:vermiculite:live mycorrhizal inoculation = 2:2:1) grown under natural light with an 18-h photoperiod. Corn cultures were assessed before using and consistently exhibited root colonization rates greater than 50% of root length colonized. 76R and *rmc* mutants were planted into nonsterilized AM pots (+AM). M82 plants were planted into nonsterilized AM pots (+AM) or into AM pots that were sterilized (−AM) by autoclaving at 121 °C for 75 min. To reintroduce any other microbes presented in +AM pots, a soil filtrate was created by mixing 2 +AM pots with 1 L of Milli-Q water. The water layer was carefully tipped off from the mixture after sedimentation. The soil filtrate was passed through a micro-cloth with a pore size of 20 μm using a vacuum to remove any AM fungal spores from the filtrate and 20 mL of this filtrate was applied to −AM pots at the time of transplantation. Tomato plants were supplied with a modified Long Ashton nutrient solution (Hewitt 1966) with 3.7 mM KNO₃ and 0.15 mM NaH₂PO₄ twice a week after transplanting, at a volume of 40 mL per pot per application. Tomato was used for hydraulic experiments 4 to 5 wk after transplanting.

Pea studies were carried out with wild-type Frisson and corresponding nonmycorrhizal *sym19* mutant. Frisson forms AM symbioses in the presence of AM fungi and the *sym19* line is mutated in *SYMRK/DMI2* gene, which encodes a leucine-rich repeat receptor-like kinase essential for activating the signaling pathway required to establish nodulation and AM symbiosis (Stracke et al. 2002). Thus, *sym19* is unable to form nodules or AM symbiosis even in the presence of rhizobia or AMF (Geurts and Bisseling 2002; Bersoult et al. 2005). Pea seeds were grown in 0.5-L pots with potting mix (potting mix:live mycorrhizal inoculum as outlined above, 4:1). Pea was provided with a modified Long Ashton nutrient solution (Hewitt 1966) with 4.8 mM KNO₃, 2.6 mM Ca(NO_3_)_2_·4H_2_O, and 0.05 mM NaH₂PO₄ once a week, with 40 mL of solution applied per pot each time. All the plants were grown in high-intensity light cabinet (*c.* 200 lm m^−2^) at 25/22 °C day/night with an 18-h photoperiod. Pea plants were used for hydraulic experiments 3 to 4 wk after planting.

### *K*_r_ in pea and tomato under well-hydrated conditions

*K*_r_ in well-watered plants (with soils at field capacity) was measured using the evaporative flux method under steady-state conditions in well-watered, unstressed plants as described in Equation (1) (Tsuda and Tyree 2000):


(1) $$K_{r}=E/(\Psi_{\mathrm{soil}}-\Psi_{\mathrm{stem}})$$

*E* is average canopy transpiration, which was measured gravimetrically in each plant under hydrated conditions with a balance (model: VIBRA ALE-6202, 6,200 G × 0.01 G) at 30-min intervals at midday between 12:00 and 14:00 (during which time transpiration and Ψ_stem_ were at steady state in both species). This was supported by graph generated from the optical dendrometer measurements of an example plant, which showed that tissue shrinkage remained stable during midday, indicating that Ψ_stem_ was at steady state (Supplementary Figure S6). *E* was normalized by the projected whole-plant leaf area measured at the end of the experiment. Ψ_stem_ was measured with a pressure chamber immediately following *E* measurements. These measurements were taken on nontranspiring leaves that had been covered with wet paper towel and aluminium foil for at least 2 h prior to *E* measurements to ensure equilibration between leaf and stem water potential. Ψ_soil_, the soil water potential, was assumed to be close to 0 MPa because soils were watered to field capacity before measurements.

### *K*_r_ in pea and tomato in response to water stress

The response of *K*_r_ (mmol s^−1^ m^−2^ MPa^−1^) to increasing soil drying was measured under nonsteady-state conditions by examining the kinetics of Ψ_stem_ relaxation during instantaneous rewetting of the soil, thereby overcoming any soil hydraulic resistance such that only *K*_r_ remained as a limiter of rehydration kinetics. Rehydration experiments were conducted on intact plants dehydrated to different levels of water stress. Biological replicates per species and treatment ranged from 12 to 19. Water deficits were created by withholding watering for different periods of time while continuously monitoring Ψ_stem_ using optical dendrometers. Optical dendrometers were attached to petioles for tomato and tendrils for pea to continuously monitor changes in tissue width and were used as a proxy for Ψ_stem_ dynamics. A linear relationship between Ψ_stem_ and width of tomato petiole/pea tendril was consistent in all plants (Supplementary Figure S7). For each plant, the dendrometer-derived width of tomato petiole/pea tendril was calibrated against Ψ_stem_ measured periodically on covered leaves with a Scholander pressure chamber (PMS Instruments, Albany, OR, USA) once the soil began to dry. After reaching the desired water stress level and before rewatering, plants were placed in the dark at high humidity (>80%) for at least 2 to 3 h to ensure stomatal closure and allow Ψ_stem_ and Ψ_soil_ to come to equilibrium (as observed with the dendrometer). Therefore, Ψ_soil_ was assumed to be equivalent to Ψ_stem_ before rewatering (after equilibration in the dark). Then the plants were rewatered and kept under the same conditions until Ψ_stem_ recovered to maximum levels (close to 0) (Supplementary Figure S6). *K*_r_ was then determined by assuming the rehydrating plant behaved as a capacitor discharging (water potential rising to zero) through a resistor and using Equation (2) by fitting an exponential curve through the first 20 min (15 to 20 data points) of Ψ_stem_ relaxation data following rewatering as follows (Brodribb and Holbrook 2004; Bourbia et al. 2021):


(2) $$K_{r}=C_{\mathrm{plant}}*ln^{\left(\Psi_{0}/\Psi_{\mathrm{final}}\right)}/t$$

where Ψ_0_ was the initial water potential before rehydration, Ψ_final_ was the water potential after rehydration for *t* = 20 min, and *C*_plant_ is whole plant capacitance (mmol m^−2^ MPa^−1^; mmol g^−1^ MPa^−1^).

### Calculating whole-plant capacitance (*C*_plant_)

The calculation of *K*_r_ required the knowledge of whole-plant capacitance (*C*_plant_). *C*_plant_ was obtained for 8 plants for each species (4 +AM plants, 4 −AM plants) used for *K*_r_ measurements by simultaneously measuring Ψ_stem_ and whole-plant mass during a dry-down from 0 to −2 MPa. At the end of the rehydration experiment, plants were removed from the pots and their roots were gently rinsed to remove all soil. An optical dendrometer was attached to the tomato petiole and pea tendril to monitor Ψ_stem_ during drying. The roots were carefully dried with absorbent paper, then the plants were placed onto a balance (model: VIBRA ALE-6202, 6,200 G × 0.01 G) and their mass (g) and Ψ_stem_ were recorded simultaneously at 10-min intervals during drying. Plants were allowed to dry slowly in the dark until their Ψ_stem_ reached −2 MPa (∼1 d), which corresponded to the lowest water potential the plants experienced during the drying experiment for *K*ᵣ measurements. *C*_plant_ was determined from the slope of the linear relationship between Ψ_stem_ and mass measured throughout drying, and it was normalized by total plant projected leaf area (m^2^) as well as root fresh weight (g). Here, the root fresh weight of the capacitance samples was determined through a regression analysis between leaf area and root fresh weight from the rehydration samples (Supplementary Figure S8).

### AM colonization assessment at final harvest

After harvesting the plants from the experiment for *K*_r_ measurement, the roots of the experimental samples were stained with the ink (LAMY, Australia) and vinegar (v:v = 1:19), as detailed in Vierheilig et al. (1998). The root mycorrhizal colonization was assessed based on the methodology of McGonigle et al. (1990). Specifically, for each plant, 150 intersects were examined from 25 root segments selected randomly. The arbuscules, vesicles, and intraradical hyphae were assessed individually. The total mycorrhizal colonization was calculated as the percentage of intersects containing any of these fungal structures. The frequency of arbuscules was assessed based on the percentage of intersects containing arbuscules.

### Statistical analysis

Linear regressions were used to quantify the correlation between the width variation of tomato petiole or pea tendril and Ψ_stem_ for each species used for *K*_r_ measurements. To assess the effects of arbuscular mycorrhizal symbiosis and Ψ_stem_ on *K*_r_ under drought conditions, a model selection was applied using the MuMIn package in R software (version 3.5.3; R Core Team 2024). The dredge function was used to generate a set of models, ranking them based on delta Akaike's Information Criterion corrected (ΔAICc) for small sample sizes with the formula as follows:


(3) $$K_{r}∼(-\Psi_{\mathrm{stem}})+\mathrm{group}+(-\Psi_{\mathrm{stem}})*\mathrm{group}$$

where group represents different treatments in each separate experiment (wild-type tomato cultivar M82 +/−AM; wild-type tomato cultivar 76R/nonmycorrhizal tomato mutant *rmc*; wild-type pea cultivar Frisson/nonmycorrhizal pea mutant *sym19*). The best models selected for each normalization method in tomato and pea were identified based on the lowest AICc values, with models having ΔAICc ≤ 2 considered to have substantial support (Supplementary Table S1) (Symonds and Moussalli 2011). This approach allowed for a comparison of multiple models that included different combinations of −Ψ_stem_, group (mycorrhizal status), and their interaction, ensuring the best model is selected based on their relative explanatory power.

Under hydrated conditions, the normality of *K*_r_ dataset was assessed for each genotype, both with and without mycorrhizal colonization. If both groups followed a normal distribution, independent *t*-tests were used to compare *K*_r_ between mycorrhizal and nonmycorrhizal plants. For any group that deviated from normality, Mann–Whitney *U* tests were applied instead. Similarly, *C*_plant_ and physiological parameters, including projected leaf area, shoot fresh weight, root fresh weight, and ratio of shoot-to-root, were compared using either *t*-tests or Mann–Whitney *U* tests, depending on the outcome of normality assessments. All statistical analyzes were performed using R software (version 3.5.3; R Core Team 2024).

## Supplementary Material

kiaf669_Supplementary_Data

## Data Availability

The data support the findings of this study are available from the corresponding author upon reasonable request.

## Supplementary material

The following materials are available in the online version of this article.

**Supplementary Figure S1.** Box plots of physiological parameters of *Solanum lycopersicum* L. and *Pisum sativum* L. plants colonized by arbuscular mycorrhiza (+AM) or with no colonization with AM (-AM).

**Supplementary Figure S2.** Relationship between the percentage of total root colonized by arbuscular mycorrhiza (AM) and root hydraulic conductance (*K*_*r*_) normalized by projected leaf area across three genotypes in *Solanum lycopersicum* L. and *Pisum sativum* L.

**Supplementary Figure S3.** Relationship between the percentage of the root colonized by arbuscules and root hydraulic conductance (*K*_*r*_) normalized by root fresh weight across three genotypes.

**Supplementary Figure S4.** Relationship between the percentage of total root colonized by arbuscular mycorrhiza (AM) and root hydraulic conductance (*K*_*r*_) normalized by root fresh weight across three genotypes in *Solanum lycopersicum* L. and *Pisum sativum* L.

**Supplementary Figure S5.** Box plots of capacitance normalized by projected leaf area and root fresh weight across genotypes in *Solanum lycopersicum* L. and *Pisum sativum* L.

**Supplementary Figure S6.** Changes in petiole width and stem water potential (*Ψ*_*stem*_) dynamics using optical dendrometers in *Solanum lycopersicum* L. during soil drying and rehydration.

**Supplementary Figure S7.** Calibration linear regression between stem water potential (-*Ψ*_*stem*_) and petiole width (in pixels) from optical dendrometer measurements in *Solanum lycopersicum* L. and *Pisum sativum* L.

**Supplementary Figure S8.** Relationship between projected leaf area and root fresh weight in *Solanum lycopersicum* L. and *Pisum sativum* L.

**Table S1.** Model selection results for analyzing root hydraulic conductance (*K*_*r*_) normalized by leaf area and root fresh weight in response to stem water potential (-*Ψ*_*stem*_) in different genotypes of *Solanum lycopersicum* L. and *Pisum sativum* L.

## References

[kiaf669-B1] Abdalla M, Ahmed MA. Arbuscular mycorrhiza symbiosis enhances water status and soil-plant hydraulic conductance under drought. Front Plant Sci. 2021:12:722954. 10.3389/fpls.2021.722954. 34721455 PMC8551442

[kiaf669-B2] Abdalla M, Bitterlich M, Jansa J, Püschel D, Ahmed MA. The role of arbuscular mycorrhizal symbiosis in improving plant water status under drought. J Exp Bot. 2023:74:4808–4824. 10.1093/jxb/erad249. 37409696

[kiaf669-B3] Albrecht C, Geurts R, Bisseling T. Legume nodulation and mycorrhizae formation; two extremes in host specificity meet. EMBO J. 1999:18:281–288. 10.1093/emboj/18.2.281. 9889184 PMC1171122

[kiaf669-B4] Allen MF . Influence of vesicular-arbuscular mycorrhizae on water movement through *Bouteloua gracilis* (HBK) Lag ex Steud. New Phytol. 1982:91:191–196. 10.1111/j.1469-8137.1982.tb03305.x.

[kiaf669-B5] Augé RM, Toler HD, Saxton AM. Mycorrhizal stimulation of leaf gas exchange in relation to root colonization, shoot size, leaf phosphorus and nitrogen: a quantitative analysis of the literature using meta-regression. Front Plant Sci. 2016:7:1084. 10.3389/fpls.2016.01084. 27524989 PMC4965464

[kiaf669-B6] Balestrini R et al Transcriptomic responses to water deficit and nematode infection in mycorrhizal tomato roots. Front Microbiol. 2019:10:1807. 10.3389/fmicb.2019.01807. 31456765 PMC6700261

[kiaf669-B7] Bersoult A et al Expression of the *Medicago truncatula* DMI2 gene suggests roles of the symbiotic nodulation receptor kinase in nodules and during early nodule development. Mol Plant Microbe Interact. 2005:18:869–876. 10.1094/MPMI-18-0869. 16134899

[kiaf669-B8] Bitterlich M, Franken P, Graefe J. Arbuscular mycorrhiza improves substrate hydraulic conductivity in the plant available moisture range under root growth exclusion. Front Plant Sci. 2018a:9:301. 10.3389/fpls.2018.00301. 29563923 PMC5845879

[kiaf669-B9] Bitterlich M, Sandmann M, Graefe J. Arbuscular mycorrhiza alleviates restrictions to substrate water flow and delays transpiration limitation to stronger drought in tomato. Front Plant Sci. 2018b:9:154. 10.3389/fpls.2018.00154. 29503655 PMC5820414

[kiaf669-B10] Boomsma CR, Vyn TJ. Maize drought tolerance: potential improvements through arbuscular mycorrhizal symbiosis? Field Crops Res. 2008:108:14–31. 10.1016/j.fcr.2008.03.002.

[kiaf669-B11] Bourbia I, Lucani C, Brodribb TJ. Constant hydraulic supply enables optical monitoring of transpiration in a grass, a herb, and a conifer. J Exp Bot. 2022:73:5625–5633. 10.1093/jxb/erac241. 35727898 PMC9467656

[kiaf669-B12] Bourbia I, Pritzkow C, Brodribb TJ. Herb and conifer roots show similar high sensitivity to water deficit. Plant Physiol. 2021:186:1908–1918. 10.1093/plphys/kiab207. 34618104 PMC8331161

[kiaf669-B13] Bourbia I, Yates LA, Brodribb TJ. Using long-term field data to quantify water potential regulation in response to VPD and soil moisture in a conifer tree. New Phytol. 2025:246:911–923. 10.1111/nph.70056. 40079639 PMC11982795

[kiaf669-B14] Brodribb TJ, Holbrook NM. Stomatal protection against hydraulic failure: a comparison of coexisting ferns and angiosperms. New Phytol. 2004:162:663–670. 10.1111/j.1469-8137.2004.01060.x. 33873766

[kiaf669-B15] Brundrett MC, Tedersoo L. Evolutionary history of mycorrhizal symbioses and global host plant diversity. New Phytol. 2018:220:1108–1115. 10.1111/nph.14976. 29355963

[kiaf669-B16] Cheng S et al Elucidating the mechanisms underlying enhanced drought tolerance in plants mediated by arbuscular mycorrhizal fungi. Front Microbiol. 2021:12:809473. 10.3389/fmicb.2021.809473. 35003041 PMC8733408

[kiaf669-B17] Chitarra W et al Insights on the impact of arbuscular mycorrhizal symbiosis on tomato tolerance to water stress. Plant Physiol. 2016:171:1009–1023. 10.1104/pp.16.00307. 27208301 PMC4902612

[kiaf669-B18] Das S, Sarkar S. Arbuscular mycorrhizal fungal contribution towards plant resilience to drought conditions. Front Fungal Biol. 2024:5:1355999. 10.3389/ffunb.2024.1355999. 38434188 PMC10904651

[kiaf669-B19] Enstone DE, Peterson CA, Ma F. Root endodermis and exodermis: structure, function, and responses to the environment. J Plant Growth Regul. 2002:21:335–351. 10.1007/s00344-003-0002-2.

[kiaf669-B20] Fang Y, Xiong L. General mechanisms of drought response and their application in drought resistance improvement in plants. Cell Mol Life Sci. 2015:72:673–689. 10.1007/s00018-014-1767-0. 25336153 PMC11113132

[kiaf669-B21] Franke R, Schreiber L. Suberin—a biopolyester forming apoplastic plant interfaces. Curr Opin Plant Biol. 2007:10:252–259. 10.1016/j.pbi.2007.04.004. 17434790

[kiaf669-B22] Geurts R, Bisseling T. Rhizobium nod factor perception and signalling. Plant Cell. 2002:14:S239–S249. 10.1105/tpc.002451. 12045280 PMC151258

[kiaf669-B23] Gonzalez-Dugo V . The influence of arbuscular mycorrhizal colonization on soil–root hydraulic conductance in *Agrostis stolonifera* L. under two water regimes. Mycorrhiza. 2010:20:365–373. 10.1007/s00572-009-0294-6. 20049617

[kiaf669-B24] Graham J, Syvertsen J. Influence of vesicular–arbuscular mycorrhiza on the hydraulic conductivity of roots of two citrus rootstocks. New Phytol. 1984:97:277–284. 10.1111/j.1469-8137.1984.tb04132.x.

[kiaf669-B25] Hewitt EJ . Sand and and water culture: methods used in the study of plant nutrition. 2nd edn. Farnham Royal, UK: Commonwealth Agricultural Bureau; 1966.

[kiaf669-B26] Huang D et al Arbuscular mycorrhizal fungi enhanced drought resistance in apple by regulating genes in the MAPK pathway. Plant Physiol Biochem. 2020:149:245–255. 10.1016/j.plaphy.2020.02.020. 32087536

[kiaf669-B27] Jeanguenin L, Mir AP, Chaumont F. Uptake, loss and control. Elsevier Inc.; 2016.

[kiaf669-B28] Kakouridis A et al Routes to roots: direct evidence of water transport by arbuscular mycorrhizal fungi to host plants. New Phytol. 2022:236:210–221. 10.1111/nph.18281. 35633108 PMC9543596

[kiaf669-B29] Kaur R, et al Hormonal regulation of drought stress in plants. Water stress and crop plants: a sustainable approach. 2016:2, John Wiley & Sons, Ltd; p. 582–599.

[kiaf669-B30] Khalvati M, Hu Y, Mozafar A, Schmidhalter U. Quantification of water uptake by arbuscular mycorrhizal hyphae and its significance for leaf growth, water relations, and gas exchange of barley subjected to drought stress. Plant Biol (Stuttg). 2005:7:706–712. 10.1055/s-2005-872893. 16388474

[kiaf669-B31] Knipfer T, Steudle E. Root hydraulic conductivity measured by pressure clamp is substantially affected by internal unstirred layers. J Exp Bot. 2008:59:2071–2084. 10.1093/jxb/ern064. 18420591 PMC2413288

[kiaf669-B32] Kuyper TW, Wang X, Muchane MN. The interplay between roots and arbuscular mycorrhizal fungi influencing water and nutrient acquisition and use efficiency. The root systems in sustainable agricultural intensification. John Wiley & Sons Ltd.; p. 193–220.

[kiaf669-B33] Levy Y, Syvertsen J, Nemec S. Effect of drought stress and vesicular–arbuscular mycorrhiza on citrus transpiration and hydraulic conductivity of roots. New Phytol. 1983:93:61–66. 10.1111/j.1469-8137.1983.tb02692.x.

[kiaf669-B34] Li Q-M, Liu B-B. Comparison of three methods for determination of root hydraulic conductivity of maize (*Zea mays* L.) root system. Agric Sci China. 2010:9:1438–1447. 10.1016/S1671-2927(09)60235-2.

[kiaf669-B35] Lim CW, Baek W, Jung J, Kim J-H, Lee SC. Function of ABA in stomatal defense against biotic and drought stresses. Int J Mol Sci. 2015:16:15251–15270. 10.3390/ijms160715251. 26154766 PMC4519898

[kiaf669-B36] Maurel C et al Aquaporins in plants. Physiol Rev. 2015:95:1321–1358. 10.1152/physrev.00008.2015. 26336033

[kiaf669-B37] McGonigle T. P., Miller M. H., Evans D. G., Fairchild G. L., Swan J. A.. A new method which gives an objective measure of colonization of roots by vesicular—arbuscular mycorrhizal fungi. New Phytologist. 1990:115(3):495–501. 10.1111/j.1469-8137.1990.tb00476.x 33874272

[kiaf669-B38] Mclean EH, Ludwig M, Grierson PF. Root hydraulic conductance and aquaporin abundance respond rapidly to partial root-zone drying events in a riparian *Melaleuca* species. New Phytol. 2011:192:664–675. 10.1111/j.1469-8137.2011.03834.x. 21848988

[kiaf669-B39] Mendes R, Garbeva P, Raaijmakers JM. The rhizosphere microbiome: significance of plant beneficial, plant pathogenic, and human pathogenic microorganisms. FEMS Microbiol Rev. 2013:37:634–663. 10.1111/1574-6976.12028. 23790204

[kiaf669-B40] Miller R, Jastrow J. Mycorrhizal fungi influence soil structure. Arbuscular mycorrhizas: physiology and function. Kluwer Academic; p. 3–18.

[kiaf669-B41] Naikwade PV . Plant responses to drought stress: morphological, physiological, molecular approaches, and drought resistance. Plant metabolites under environmental stress. Apple Academic Press; p. 149–183.

[kiaf669-B42] Obase K, Kitagami Y, Tanikawa T, Chen C-F, Matsuda Y. Fungi and bacteria in the rhizosphere of *Cryptomeria japonica* exhibited different community assembly patterns at regional scales in East Asia. Rhizosphere. 2023:28:100807. 10.1016/j.rhisph.2023.100807.

[kiaf669-B43] Omotayo OP, Babalola OO. Resident rhizosphere microbiome’s ecological dynamics and conservation: towards achieving the envisioned sustainable development goals, a review. Int Soil Water Conserv Res. 2021:9:127–142. 10.1016/j.iswcr.2020.08.002.

[kiaf669-B44] Pauwels R, Graefe J, Bitterlich M. An arbuscular mycorrhizal fungus alters soil water retention and hydraulic conductivity in a soil texture specific way. Mycorrhiza. 2023:33:165–179. 10.1007/s00572-023-01106-8. 36976365 PMC10244285

[kiaf669-B45] Prado K, Maurel C. Regulation of leaf hydraulics: from molecular to whole plant levels. Front Plant Sci. 2013:4:255. 10.3389/fpls.2013.00255. 23874349 PMC3711007

[kiaf669-B46] Prihatna C, Larkan NJ, Barbetti MJ, Barker SJ. Tomato CYCLOPS/IPD3 is required for mycorrhizal symbiosis but not tolerance to *Fusarium* wilt in mycorrhiza-deficient tomato mutant rmc. Mycorrhiza. 2018:28:495–507. 10.1007/s00572-018-0842-z. 29948410

[kiaf669-B47] Püschel D, Bitterlich M, Rydlová J, Jansa J. Facilitation of plant water uptake by an arbuscular mycorrhizal fungus: a Gordian knot of roots and hyphae. Mycorrhiza. 2020:30:299–313. 10.1007/s00572-020-00949-9. 32253570

[kiaf669-B48] Püschel D, Bitterlich M, Rydlová J, Jansa J. Drought accentuates the role of mycorrhiza in phosphorus uptake. Soil Biol Biochem. 2021:157:108243. 10.1016/j.soilbio.2021.108243.

[kiaf669-B49] Qin M et al Experimental duration determines the effect of arbuscular mycorrhizal fungi on plant biomass in pot experiments: a meta-analysis. Front Plant Sci. 2022:13:1024874. 10.3389/fpls.2022.1024874. 36407631 PMC9671359

[kiaf669-B50] Rengel Z, Djalovic I. The root systems in sustainable agricultural intensification. Wiley Online Library.

[kiaf669-B51] Rodriguez-Dominguez CM, Brodribb TJ. Declining root water transport drives stomatal closure in olive under moderate water stress. New Phytol. 2020:225:126–134. 10.1111/nph.16177. 31498457

[kiaf669-B52] Ruiz-Lozano JM et al Arbuscular mycorrhizal symbiosis induces strigolactone biosynthesis under drought and improves drought tolerance in lettuce and tomato. Plant Cell Environ. 2016:39:441–452. 10.1111/pce.12631. 26305264

[kiaf669-B53] Ruiz-Lozano JM, Azcon R, Gomez M. Effects of arbuscular-mycorrhizal *Glomus* species on drought tolerance: physiological and nutritional plant responses. Appl Environ Microbiol. 1995:61:456–460. 10.1128/aem.61.2.456-460.1995. 16534929 PMC1388347

[kiaf669-B54] Steudle E . Water uptake by roots: effects of water deficit. J Exp Bot. 2000:51:1531–1542. 10.1093/jexbot/51.350.1531. 11006304

[kiaf669-B55] Stracke S et al A plant receptor-like kinase required for both bacterial and fungal symbiosis. Nature. 2002:417:959–962. 10.1038/nature00841. 12087405

[kiaf669-B56] Symonds MR, Moussalli A. A brief guide to model selection, multimodel inference and model averaging in behavioural ecology using Akaike’s information criterion. Behav Ecol Sociobiol. 2011:65:13–21. 10.1007/s00265-010-1037-6.

[kiaf669-B57] Tsuda M, Tyree MT. Plant hydraulic conductance measured by the high pressure flow meter in crop plants. J Exp Bot. 2000:51:823–828. 10.1093/jexbot/51.345.823. 10938875

[kiaf669-B58] Ullah A et al Phosphorous supplementation alleviates drought-induced physio-biochemical damages in *Calligonum mongolicum*. Plants. 2022:11:3054. 10.3390/plants11223054. 36432784 PMC9699272

[kiaf669-B59] Vadez V . Root hydraulics: the forgotten side of roots in drought adaptation. Field Crops Res. 2014:165:15–24. 10.1016/j.fcr.2014.03.017.

[kiaf669-B60] Vierheilig H, Coughlan A, Wyss U, Piché Y. Ink and Vinegar, a Simple Staining Technique for Arbuscular-Mycorrhizal Fungi. Appl Environ Microbiol. 1998:64(12):5004–5007. 10.1128/AEM.64.12.5004-5007.1998 9835596 PMC90956

[kiaf669-B61] Wahab A et al Role of arbuscular mycorrhizal fungi in regulating growth, enhancing productivity, and potentially influencing ecosystems under abiotic and biotic stresses. Plants (Basel). 2023:12(17):3102. 10.3390/plants12173102. 37687353 PMC10489935

[kiaf669-B62] Wang Y, Zou Y-N, Shu B, Wu Q-S. Deciphering molecular mechanisms regarding enhanced drought tolerance in plants by arbuscular mycorrhizal fungi. Sci Hortic. 2023:308:111591. 10.1016/j.scienta.2022.111591.

[kiaf669-B63] Xie W et al Integrated transcriptomics and metabolomics reveal specific phenolic and flavonoid accumulation in licorice (*Glycyrrhiza uralensis* Fisch.) induced by arbuscular mycorrhiza symbiosis under drought stress. Plant Physiol Biochem. 2023:205:108173. 10.1016/j.plaphy.2023.108173. 37984021

[kiaf669-B64] Xu L et al Arbuscular mycorrhiza enhances drought tolerance of tomato plants by regulating the 14-3-3 genes in the ABA signaling pathway. Appl Soil Ecol. 2018:125:213–221. 10.1016/j.apsoil.2018.01.012.

[kiaf669-B65] Yan Q et al Arbuscular mycorrhizal fungi improve the growth and drought tolerance of *Cinnamomum migao* by enhancing physio-biochemical responses. Ecol Evol. 2022:12:e9091. 10.1002/ece3.9091. 35845374 PMC9273509

[kiaf669-B66] Yang X et al How do arbuscular mycorrhizal fungi enhance drought resistance of *Leymus chinensis*? BMC Plant Biol. 2025:25:1–14. 10.1186/s12870-025-06412-1. 40211145 PMC11984051

[kiaf669-B67] Yano K et al CYCLOPS, a mediator of symbiotic intracellular accommodation. Proc Natl Acad Sci U S A. 2008:105:20540–20545. 10.1073/pnas.0806858105. 19074278 PMC2629324

[kiaf669-B68] Zhang Q, Zhang L, Weiner J, Tang J, Chen X. Arbuscular mycorrhizal fungi alter plant allometry and biomass–density relationships. Ann Bot. 2011:107:407–413. 10.1093/aob/mcq249. 21169608 PMC3043928

[kiaf669-B69] Zhang Z, Zhang J, Xu G, Zhou L, Li Y. Arbuscular mycorrhizal fungi improve the growth and drought tolerance of *Zenia insignis* seedlings under drought stress. New For (Dordr). 2019:50:593–604. 10.1007/s11056-018-9681-1.

[kiaf669-B70] Zou Y-N, Wu H-H, Giri B, Wu Q-S, Kuča K. Mycorrhizal symbiosis down-regulates or does not change root aquaporin expression in trifoliate orange under drought stress. Plant Physiol Biochem. 2019:144:292–299. 10.1016/j.plaphy.2019.10.001. 31600710

