# Supplementary material for: Arbuscular mycorrhizal colonization does not improve root hydraulic supply in tomato and pea
Source: Plant Physiol. 2025 Dec 23;200(1):kiaf669. doi: 10.1093/plphys/kiaf669 (PMC12854237; doi:10.1093/plphys/kiaf669)
Supplement: kiaf669_Supplementary_Data [file kiaf669_supplementary_data.pdf]

SUPPLEMENTARY FIGURES

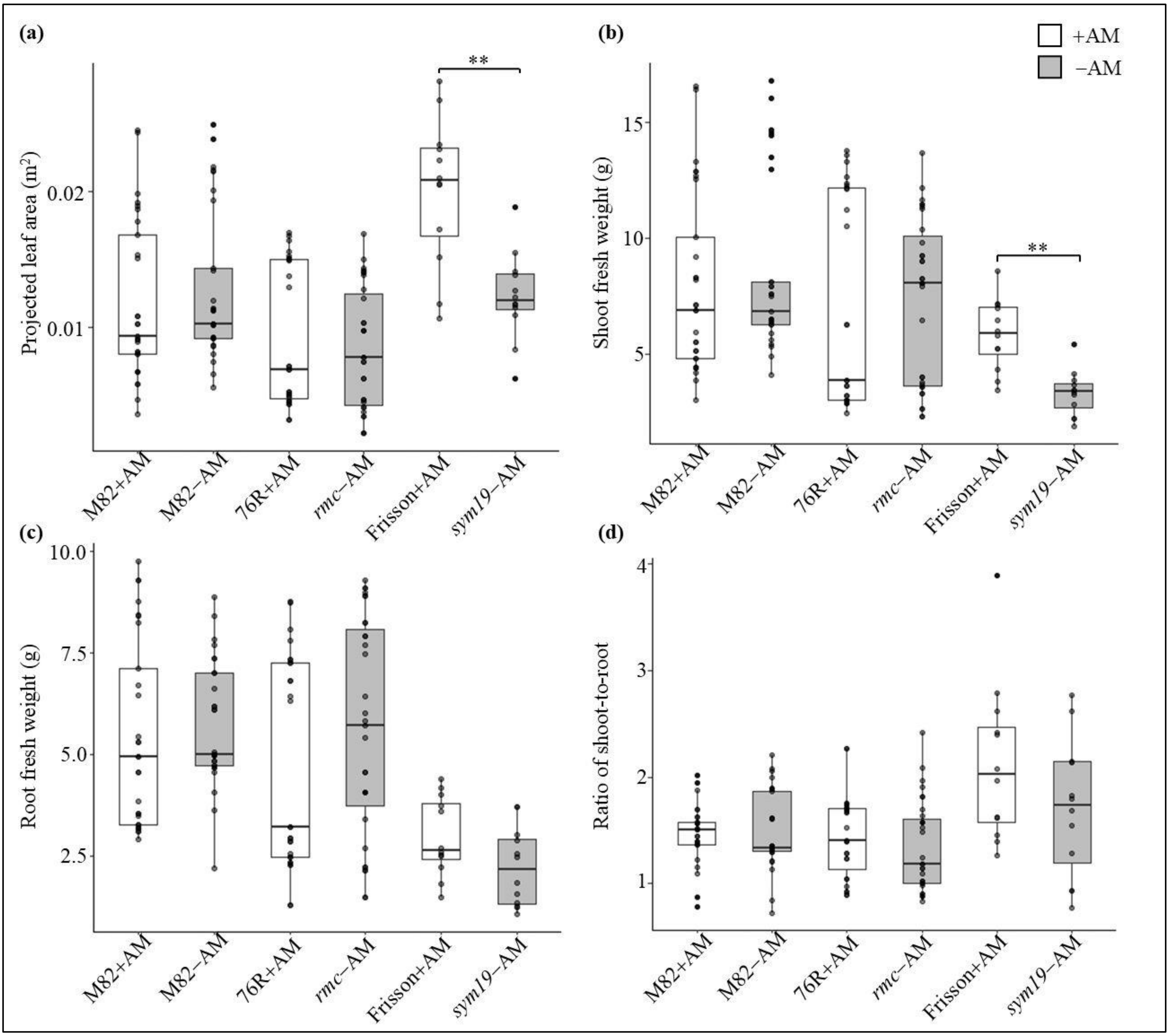

Fig. S1 Box plots of physiological parameters of *Solanum lycopersicum* L. and *Pisum sativum* L. plants colonized by arbuscular mycorrhiza (+AM) or with no colonization with AM (−AM). (a) Projected leaf area (m^2), **(b)** Shoot fresh weight (g), **(c)** Root fresh weight (g), and **(d)** Ratio of shoot and root. Tomato genotypes are M82 (wild type), 76R (wild type) and respective non-mycorrhizal mutant *rmc* (n = 29-31). Pea genotypes are Frisson (wild type) and respective non-mycorrhizal mutant *sym19* (n = 12). Each data point represents individual plant replicate samples. The center line represents the median; box limits indicate the upper and lower quartiles; whiskers extend to 1.5× the interquartile range; points represent individual biological replicates, with points beyond the whiskers considered outliers. Asterisks indicate significant differences between two genotypes (t-test, **, $p < 0.01$).

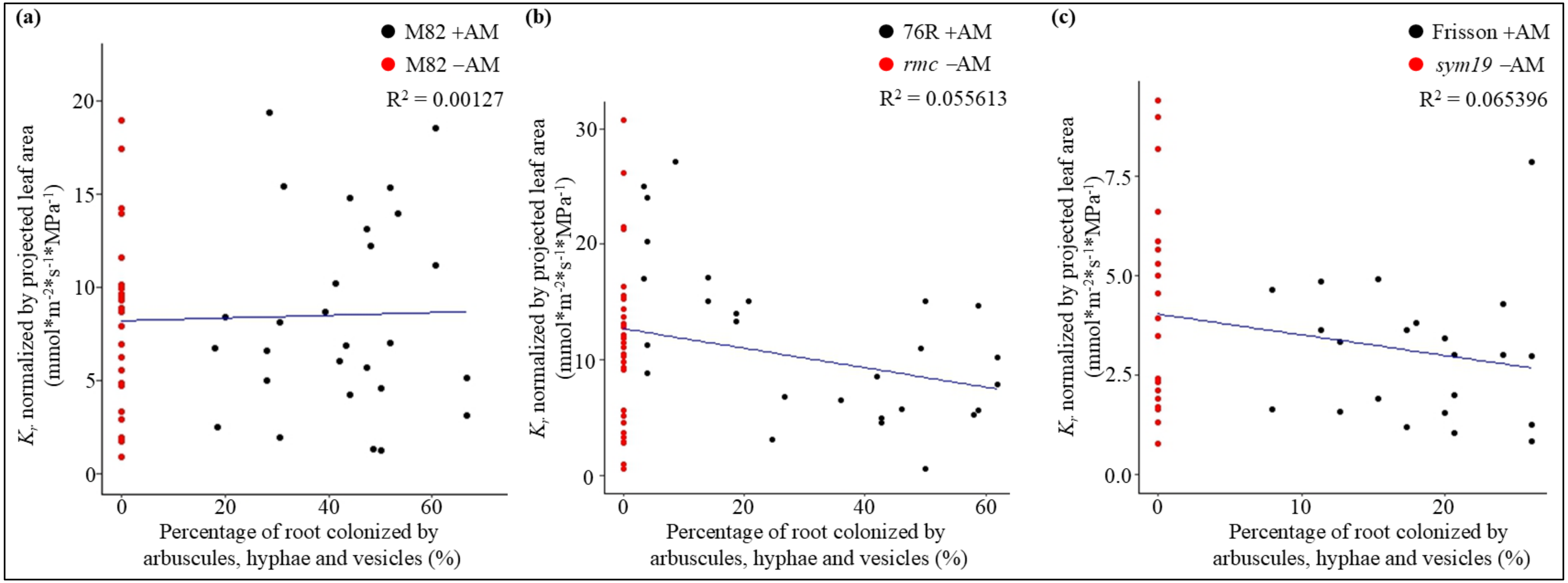

Fig. S2 Relationship between the percentage of total root colonized by arbuscular mycorrhiza (AM) and root hydraulic conductance (K_r) normalized by projected leaf area across three genotypes in *Solanum lycopersicum* L. and *Pisum sativum* L. (a) Tomato M82 (wild type), with arbuscular mycorrhiza (+AM) and without AM (−AM). **(b)** Tomato 76R (wild type) and its non-mycorrhizal mutant, *rmc*. **(c)** Pea Frisson (wild type) and its non-mycorrhizal mutant, *sym19*. R^2 values are displayed in the top-right corner to indicate the goodness of fit for each regression model.

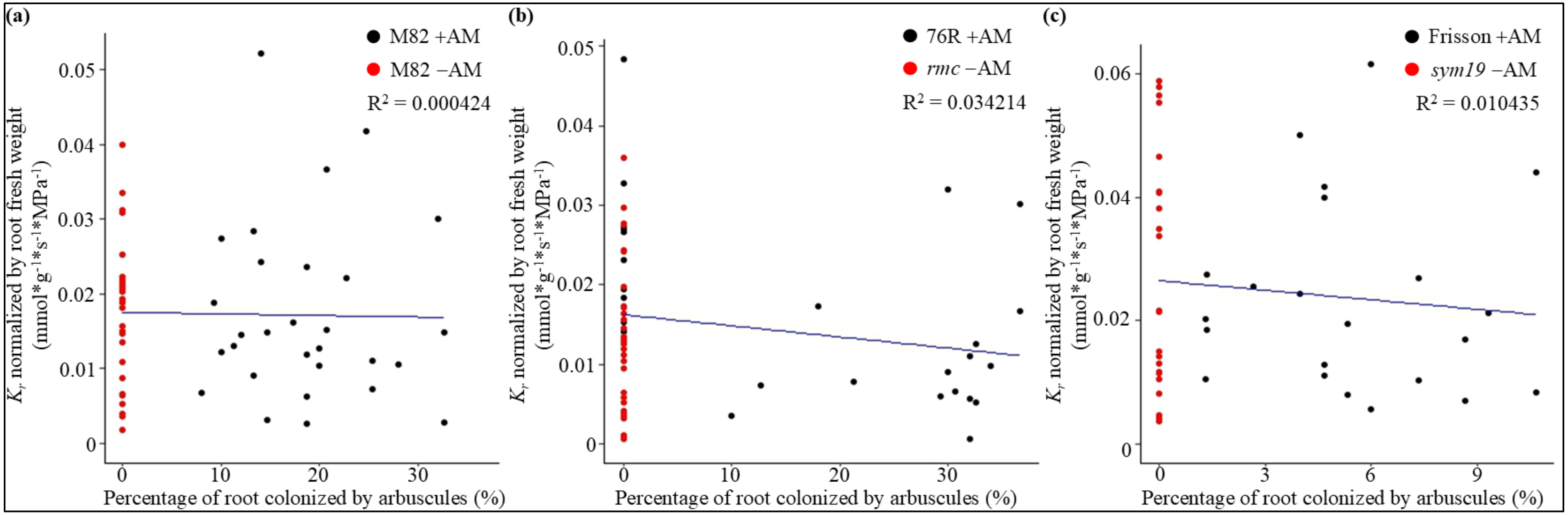

Fig. S3 Relationship between the percentage of the root colonized by arbuscules and root hydraulic conductance (K_r) normalized by root fresh weight across three genotypes in *Solanum lycopersicum* L. and *Pisum sativum* L. (a) Tomato M82 (wild type), with arbuscular mycorrhiza (+AM) and without AM (−AM). **(b)** Tomato 76R (wild type) and its non-mycorrhizal mutant, *rmc*. **(c)** Pea Frisson (wild type) and its non-mycorrhizal mutant, *sym19*. R^2 values are displayed in the top-right corner to indicate the goodness of fit for each regression model.

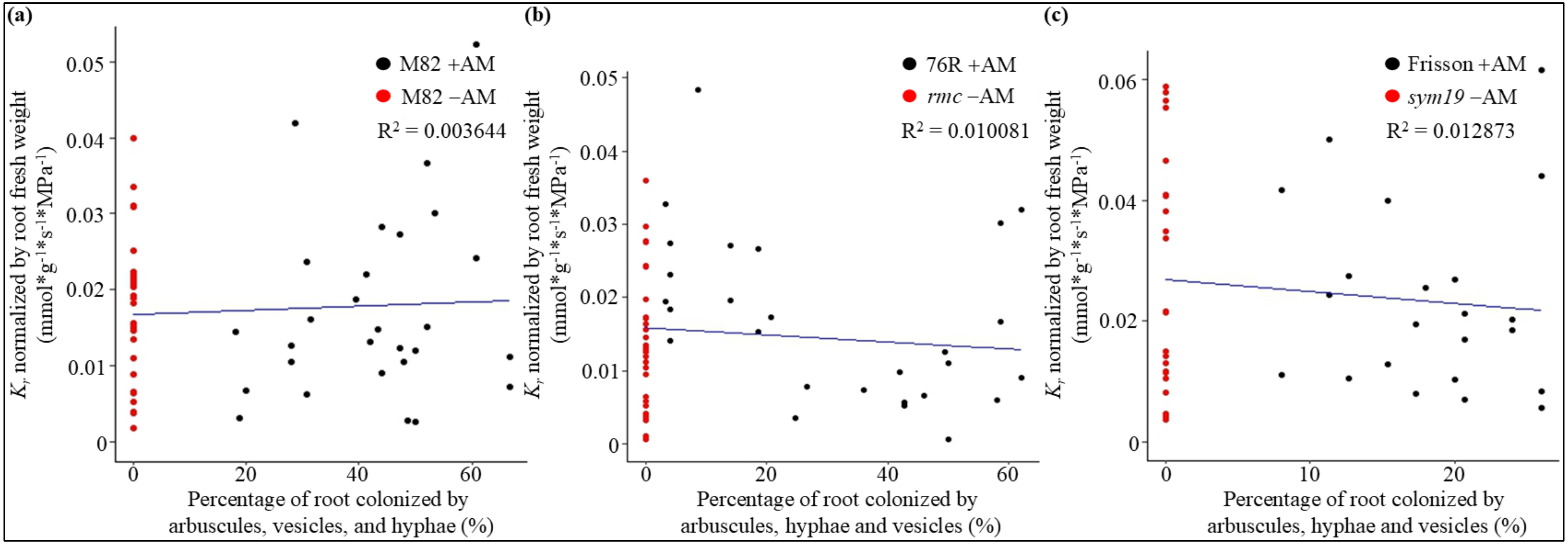

Fig. S4 Relationship between the percentage of total root colonized by arbuscular mycorrhiza (AM) and root hydraulic conductance (K_r) normalized by root fresh weight across three genotypes in *Solanum lycopersicum* L. and *Pisum sativum* L. (a) Tomato M82 (wild type), with arbuscular mycorrhiza (+AM) and without AM (−AM). (b) Tomato 76R (wild type) and its non-mycorrhizal mutant, *rmc*. (c) Pea Frisson (wild type) and its non-mycorrhizal mutant, *sym19*. R^2 values are displayed in the top-right corner to indicate the goodness of fit for each regression model.

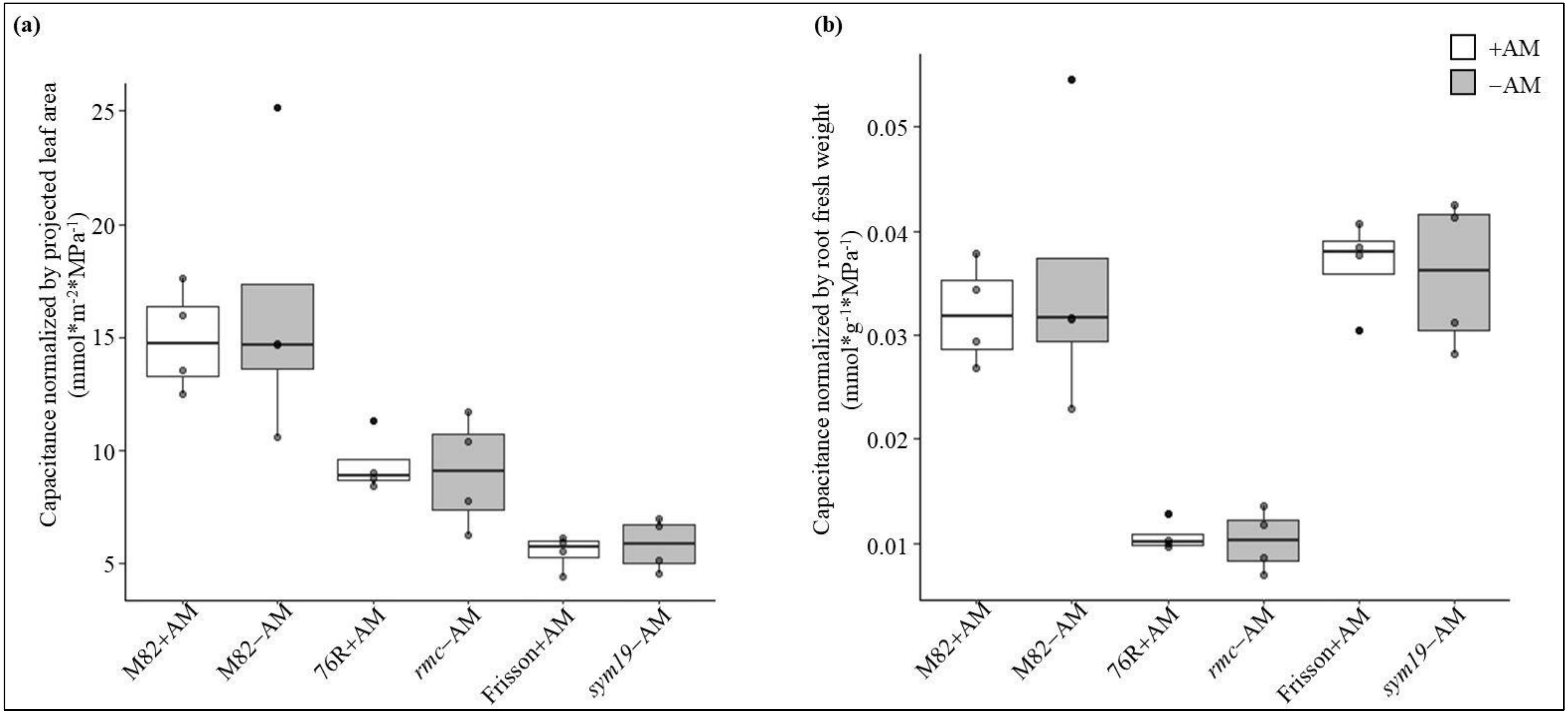

Fig. S5 Box plots of capacitance normalized by projected leaf area and root fresh weight across genotypes in *Solanum lycopersicum* L. and *Pisum sativum* L. (a) Capacitance normalized by projected leaf area (m^2); **(b)** Capacitance normalized by root fresh weight (g). Tomato genotypes are M82 (wild type), 76R (wild type) and respective non-mycorrhizal mutant *rmc* (n = 4). Pea genotypes are Frisson (wild type) and respective non-mycorrhizal mutant *sym19* (n = 4). The center line represents the median; box limits indicate the upper and lower quartiles; whiskers extend to 1.5× the interquartile range; points represent individual biological replicates, with points beyond the whiskers considered outliers. Each data point represents individual replicate samples.

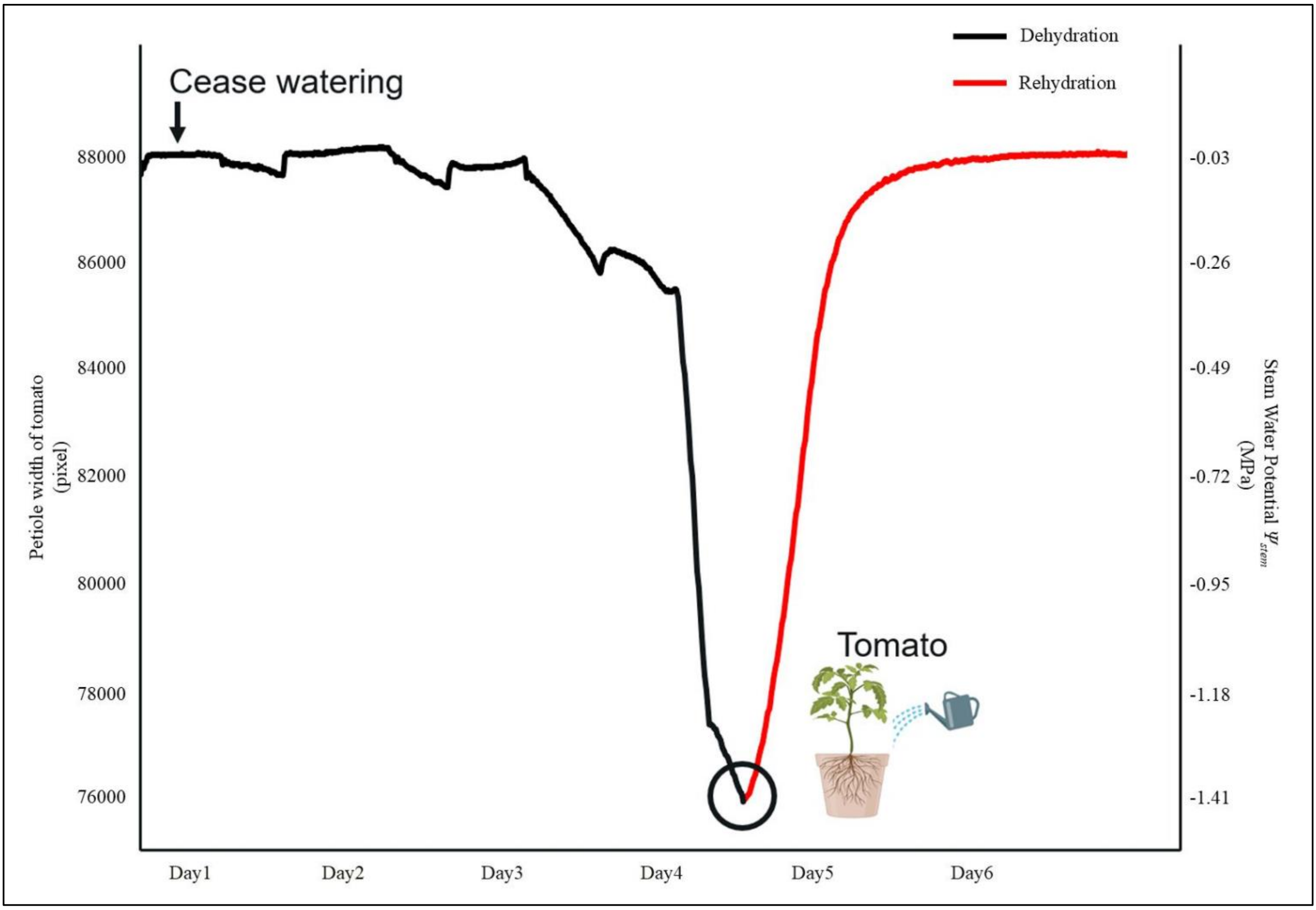

Fig. S6 Changes in petiole width and stem water potential (Ψ_{stem}) dynamics using optical dendrometers in *Solanum lycopersicum* L. during soil drying and rehydration. One replicate of M82 wild-type tomato inoculated with arbuscular mycorrhizal fungi are shown as a representative example. Watering was ceased on Day 1, as indicated by the arrow. Color-coded lines represent different stages: dehydration period (black), and recovery post-rewatering (red).

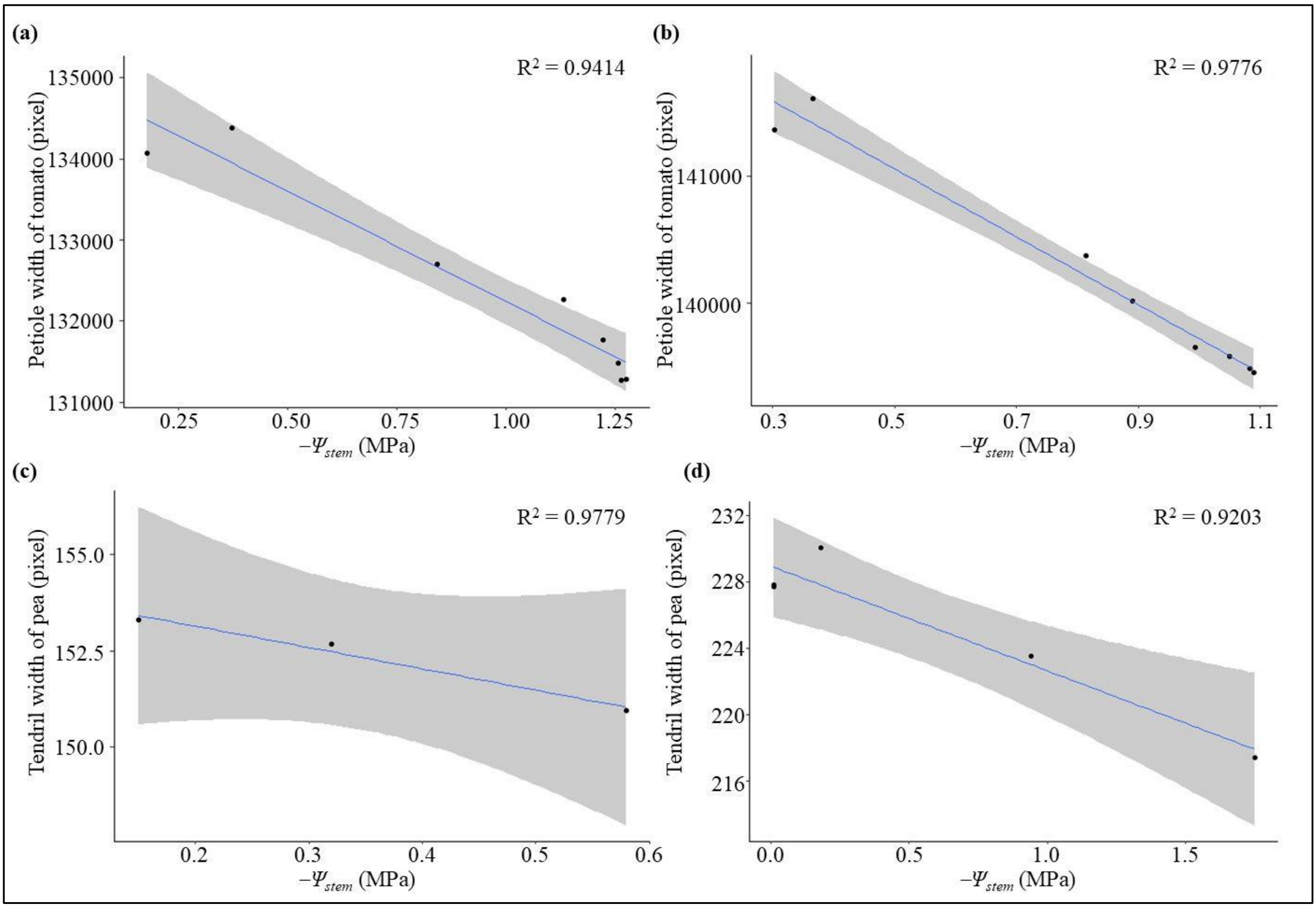

Fig. S7 Calibration linear regression between stem water potential ($-\Psi_{stem}$) and petiole width (in pixels) from optical dendrometer measurements in *Solanum lycopersicum* L. and *Pisum sativum* L. **(a)** and **(b)** show the calibration for tomato, while **(c)** and **(d)** represent the calibration for pea. The shaded area around each regression line indicates the 95% confidence interval. R^2 values are displayed in the top-right corner to indicate the goodness of fit for each regression model. Each panel represents the repeated measurements of individual plant. The sample size of tomato is n = 8; the sample size of pea is n = 3-4.

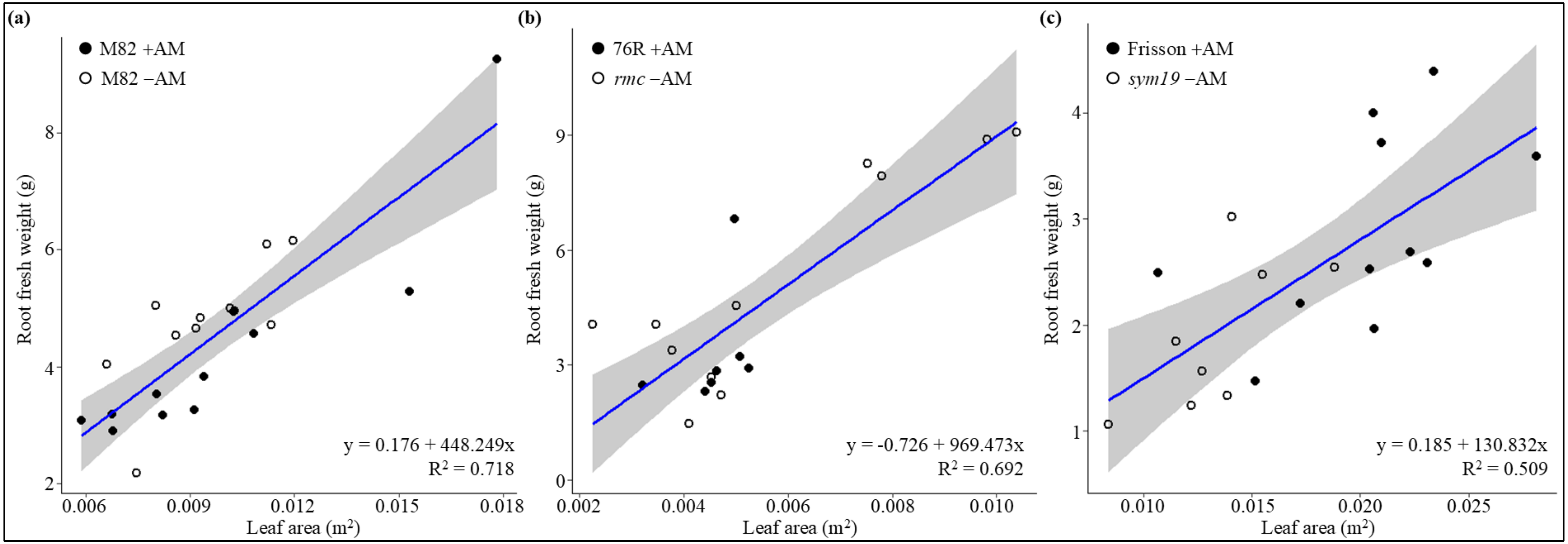

Fig. S8 Relationship between projected leaf area and root fresh weight in *Solanum lycopersicum* L. and *Pisum sativum* L. (a) Tomato wild type (M82) with arbuscular mycorrhiza (+AM) and without arbuscular mycorrhiza (–AM) (n = 22); **(b)** Tomato wild type (76R) colonized by mycorrhizal fungi (+AM) and its respective non-mycorrhizal mutant *rmc* (–AM) (n = 18); **(c)** Pea wild type (Frisson) colonized by mycorrhizal fungi (+AM) and its respective non-mycorrhizal *sym19* (–AM) (n = 19).

Table S1 Model selection results for analyzing root hydraulic conductance (K_r) normalized by leaf area and root fresh weight in response to stem water potential ($-\Psi_{stem}$) across different genotypes of *Solanum lycopersicum* L. and *Pisum sativum* L.

df: degrees of freedom

AICc: Akaike's Information Criterion corrected

ΔAICc: Difference in Akaike Information Criterion corrected for small sample sizes

Intercept: Baseline level of the response variable when all predictors are set to zero

Group: Represents the interaction between different treatments (+/−AM) in each experiment (from above to below, group represents: wild-type tomato cultivar M82+/−AM; wild-type tomato cultivar M82+/−AM; wild-type tomato cultivar 76R/non-mycorrhizal tomato mutant *rmc*; wild-type tomato cultivar 76R/non-mycorrhizal tomato mutant *rmc*; wild-type pea cultivar Frisson/non-mycorrhizal pea mutant *sym19*; wild-type pea cultivar Frisson/non-mycorrhizal pea mutant *sym19*)

NA: Not applicable

+: With significant difference

Data	Model No.	Intercept	group	$-\Psi_{stem}$	Interaction between group and $-\Psi_{stem}$	df	Log-Likelihood	AICc	ΔAICc	weight
K_r normalized by leaf area: Wild-type tomato cultivar M82+/−AM	1	12.28	NA	-5.82	NA	3	-79.14	165.1944	0.00	0.68
	2	11.87	+	-5.81	NA	4	-78.93	167.4606	2.27	0.22
K_r normalized by root fresh weight: Wild-type tomato cultivar M82+/−AM	3	0.03	NA	-0.01	NA	3	105.00	-203.0701	0.00	0.68
	4	0.03	+	-0.01	NA	4	105.21	-200.8288	2.24	0.22
K_r normalized by leaf area: Wild-type tomato cultivar 76R/non-mycorrhizal tomato mutant *rmc*	5	21.66	NA	-15.30	NA	3	-115.74	238.2047	0.00	0.73
	6	21.73	+	-15.32	NA	4	-115.74	240.7242	2.52	0.21
K_r normalized by root fresh weight: Wild-type tomato cultivar 76R/non-mycorrhizal tomato mutant *rmc*	7	0.02	NA	-0.02	NA	3	135.02	-263.316	0.00	0.73
	8	0.02	+	-0.02	NA	4	135.02	-260.7941	2.52	0.21
	9	5.08	+	-1.46	NA	4	-36.90	83.89843	0.00	0.45

K_r normalized by leaf area: Wild-type pea cultivar Frisson/non-mycorrhizal pea mutant *sym19*	10	4.76	NA	-1.52	NA	3	-38.49	84.17235	0.27	0.39
	11	4.68	+	-1.19	+	5	-36.57	86.47667	2.58	0.12
K_r normalized by root fresh weight: Wild-type pea cultivar Frisson/non-mycorrhizal pea mutant *sym19*	12	0.03	NA	-0.01	NA	3	83.80	-160.3955	0.00	0.53
	13	0.03	+	-0.01	NA	4	84.80	-159.4975	0.90	0.34
	14	0.03	+	-0.01	+	5	85.29	-157.2384	3.16	0.11
